# Transformation of nonencapsulated *Streptococcus pneumoniae* during systemic infection

**DOI:** 10.1038/s41598-020-75988-5

**Published:** 2020-11-03

**Authors:** Jessica L. Bradshaw, Iftekhar M. Rafiqullah, D. Ashley Robinson, Larry S. McDaniel

**Affiliations:** 1 Department of Microbiology and Immunology, University of Mississippi Medical Center, Jackson, MS USA; 2 Present Address: Department of Physiology and Anatomy, University of North Texas Health Science Center, Fort Worth, TX USA

**Keywords:** Bacteriology, Pathogens

## Abstract

*Streptococcus pneumoniae* (pneumococcus) is a principal cause of bacterial middle ear infections, pneumonia, and meningitis. Capsule-targeted pneumococcal vaccines have likely contributed to increased carriage of nonencapsulated *S. pneumoniae* (NESp). Some NESp lineages are associated with highly efficient DNA uptake and transformation frequencies. However, NESp strains lack capsule that may increase disease severity. We tested the hypothesis that NESp could acquire capsule during systemic infection and transform into more virulent pneumococci. We reveal that NESp strains MNZ67 and MNZ41 are highly transformable and resistant to multiple antibiotics. Natural transformation of NESp when co-administered with heat-killed encapsulated strain WU2 in a murine model of systemic infection resulted in encapsulation of NESp and increased virulence during bacteremia. Functional capsule production increased the pathogenic potential of MNZ67 by significantly decreasing complement deposition on the bacterial surface. However, capsule acquisition did not further decrease complement deposition on the relatively highly pathogenic strain MNZ41. Whole genome sequencing of select transformants demonstrated that recombination of up to 56.7 kbp length occurred at the capsule locus, along with additional recombination occurring at distal sites harboring virulence-associated genes. These findings indicate NESp can compensate for lack of capsule production and rapidly evolve into more virulent strains.

## Introduction

*Streptococcus pneumoniae* (pneumococcus) resides harmlessly in the nasopharynx of asymptomatic carriers, but is also a prominent human pathogen that consistently causes mucosal diseases of the upper respiratory system^1^. Noninvasive diseases associated with pneumococcal infections include conjunctivitis, otitis media (OM), and nonbacteremic pneumonia^2,3^. Invasive pneumococcal disease (IPD) can occur when pneumococci disseminate into normally sterile sites such as the blood and meninges to establish bacteremia and bacterial meningitis, respectively^2^. Prevention measures against pneumococcal disease have been implemented for decades and include two commercially licensed vaccines: PNEUMOVAX 23 and Prevnar 13^4–6^. Still, the pneumococcus remains a leading cause of bacterial OM, pneumonia, and meningitis even with vaccine implementation^7^. The resilient pathogenicity of pneumococcus in spite of prevention measures is largely due to limitations in the vaccine strategy and genomic plasticity of the pneumococcus. Currently, there are nearly 100 unique pneumococcal serotypes identified, and the current vaccines together target roughly 25% of the characterized serotypes^8^. Furthermore, selective pressure on vaccine-inclusive serotypes has likely driven capsule switching and positive selection of mutants expressing antigenically distinct capsule structures and of nonencapsulated *S. pneumoniae* (NESp) that allow escape from antibody-mediated clearance.

NESp cannot be characterized by capsular serotyping since this subpopulation does not express the polysaccharide capsule. Rather, NESp are characterized by the genes present in the capsular polysaccharide biosynthetic (*cps*) locus^9^. Strains that encode nonfunctional variants of capsule genes found in encapsulated strains are considered Group I NESp. Strains characterized as Group II NESp lack the genes encoded in the *cps* locus of encapsulated strains and instead encode virulence-associated genes *pspK*, *aliC*, and *aliD* in the *cps* locus. Group II NESp can be further divided into so-called null capsule clades (NCC) based on the genes present in the *cps* locus^9^. NESp strains that encode *pspK* are designated NCC1 while strains that encode *aliC* and *aliD* are considered NCC2.

The variability of the pneumococcal genome is greatly impacted by horizontal gene transfer (HGT). Pneumococci are naturally competent for transformation and are thus able to import exogenous DNA that is subsequently integrated into the chromosome via homologous recombination^10^. Integrative conjugative elements (ICEs) and phages may also be present in pneumococcal strains and provide additional mechanisms for HGT. Bacterial factors enhancing colonization and virulence, as well as antibacterial resistance and immune evasion, have been acquired through HGT^11,12^. Furthermore, NESp are associated with multidrug resistance, which may be a consequence of higher recombination frequencies observed in some NESp when compared to encapsulated pneumococci^13^. Interactions between pneumococcal strains during cocolonization of the nasopharynx provide opportunities for intraspecies HGT to occur. NESp have been shown to colonize the nasopharynx as efficiently as encapsulated pneumococci, and genetic exchange between these populations does occur^14,15^.

*S. pneumoniae* strains are diverse and express various virulence factors that potentiate mucosal and invasive infections^1,16^. The polysaccharide capsule is a major virulence factor of the pneumococcus^1^. However, the specific impact of virulence factors is dependent upon the host niche being occupied^17^. For instance, capsule has been shown to have varying impacts on disease states. During colonization of the nasopharynx or lungs and in cases of conjunctivitis, capsule expression is reduced or negatively impacts pneumococcal virulence^18,19^. Yet, capsule has also been shown to be an extremely significant virulence factor due to its protective function during invasive disease^20^. NESp colonize the nasopharynx well but are not efficient in establishing invasive disease^3,21^. Thus, capsule acquisition could expand the pathogenic capabilities of NESp.

In a classic experiment, Frederick Griffith first demonstrated that attenuated, rough colonies could be “transformed” into virulent, smooth colonies during murine infections^22^. However, Griffith used a passaged, avirulent strain rather than a naturally nonencapsulated strain as a transformation recipient. Emerging NESp strains encode novel genes and genetic elements in comparison to those of encapsulated strains^1^. Thus, virulence-associated consequences of genetic transfer and capsule acquisition in some NESp may be far greater than a null capsule mutant regaining capsule. Moreover, NESp that have acquired multiple antibiotic resistance genes may serve as antibiotic resistance reservoirs that greatly threaten treatment options and survival outcomes^3^. In this study, we hypothesized that naturally nonencapsulated strains will be efficiently transformed when under the selective pressure of invasive disease into more virulent strains. The aim of this study was to determine genetic and phenotypic alterations that occur in NESp during in vivo transformation.

## Results

### NESp strains are antibiotic resistant and highly transformable

We first determined in vitro antibiotic susceptibilities of donor and recipient pneumococcal strains used for in vivo transformations. The donor strain, encapsulated serotype 3 *S. pneumoniae* WU2, was sensitive to both erythromycin (Erm) and trimethoprim (Tmp), while recipient NESp strains MNZ41 and MNZ67 were resistant to Tmp or Erm, respectively (Table 1). These two NESp strains are of different multilocus sequence types and have different genes at the *cps* locus; thus, these strains represent different NESp lineages. Next, we evaluated in vitro transformation efficiencies of NESp recipient strains using genomic DNA isolated from *S. pneumoniae* LEK06 harboring chromosomally acquired streptomycin resistance. To become competent for transformation, MNZ67 required only competence stimulating peptide (Csp)-1 while MNZ41 required both Csp-1 and Csp-2. MNZ67 had a higher transformation efficiency (204 ± 46 transformants/µg DNA) compared to MNZ41 (164 ± 44 transformants/µg DNA), but this difference was not statistically significant (*p* = 0.593, student T-test).

**Table 1:** Description of strains used in this study.

Strain	Sequence type	Serotype	Transformation contribution	Antibiotic susceptibility	Capsule locus genes	References
WU2	378	3	Donor (in vivo)	Erm^S^ Tmp^S^	*cpsA*^+^	^46^
LEK06	43	19F	Donor (in vitro)	Str^R^	*cpsA*^+^	This study
MNZ67	1464	NESp	Recipient	Erm^R^	*cpsA*^*−*^ *pspK*^+^	^9^
67S1	1464	3	MNZ67 Transformant	Erm^R^	*cpsA*^+^ *pspK*^*−*^	This study
MNZ41	6153	NESp	Recipient	Tmp^R^	*cpsA*^*−*^ *aliC*^+^ *aliD*^+^	^9^
41S3	6153	3	MNZ41 Transformant	Tmp^R^	*cpsA*^+^ *aliC*^*−*^ *aliD*^*−*^	This study

Capsule genes were determined by polymerase chain reaction (PCR) analysis and verified by whole genome sequencing.

NESp, nonencapsulated *Streptococcus pneumoniae*; Str, streptomycin; Erm, erythromycin; TMP, trimethoprim; S, sensitive; R, resistant.

### NESp strains acquire and express capsule during systemic infection

Since MNZ67 and MNZ41 were highly transformable in vitro, we investigated transformation of these strains during a murine model of systemic infection. Heat-killed *S. pneumoniae* strain WU2 was chosen as a DNA donor because of its mucoid capsule phenotype on blood agar plates that would permit the identification of transformants in a mixed culture of nonencapsulated and encapsulated pneumococci. In mice administered an NESp strain and heat-killed WU2 (ΔWU2), no capsule transformants were identified in blood mixtures isolated at 4 h postinfection (hpi), as all isolated pneumococci displayed a rough phenotype characteristic of nonencapsulated strains. At 24 hpi, smooth transformants were isolated from mice administered either NESp parent strain with ΔWU2, indicating that in vivo pneumococcal transformation occurs within the first 24 h. At 24 hpi, homogenous mixtures of nonencapsulated or encapsulated pneumococci, as well as heterogenous mixtures containing both rough and smooth phenotypes, were isolated from the blood of individual mice administered NESp strains and ΔWU2 (Fig. S1). Two in vivo capsule transformants, 41S3 and 67S1, were selected for screening based on their smooth phenotype on blood agar plates. The genetic basis of this phenotype was initially examined by PCR analysis of the capsular locus. All isolates that appeared to express capsule on blood agar encoded the conserved *cpsA* gene that replaced *pspk* or *aliC* and *aliD* encoded in the respective parent strains (Table 1). Furthermore, in vivo transformants that encoded the conserved capsule gene *cpsA* also retained antibiotic resistance associated with parent (recipient) strains (Table 1).

### Capsule acquisition enhances persistence and virulence of transformed NESp during bacteremia

**Figure 1:**
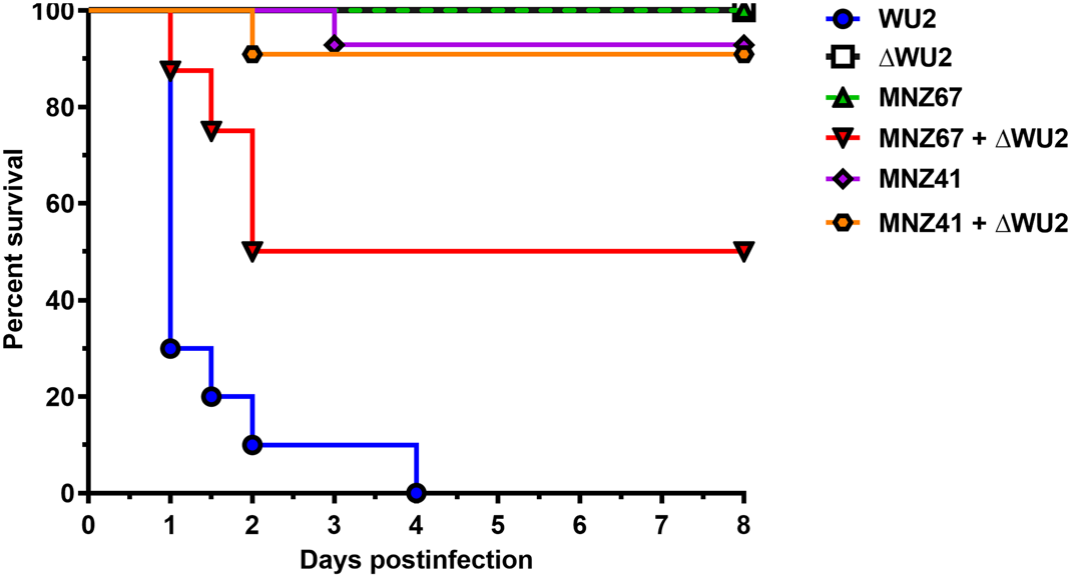
Survival analysis of C57BL/6 mice during pneumococcal bacteremia. Seven-week-old mice were intraperitoneally infected with indicated pneumococcal strains, and survival was monitored over an 8-day period. Survival rates are shown as percentages (WU2: n = 10, ΔWU2: n = 6, MNZ67: n = 8, MNZ67 + ΔWU2: n = 8, MNZ41: n = 14, MNZ41 + ΔWU2: n = 11). Significantly higher mortality rates were observed in mice infected with WU2 or MNZ67 + ΔWU2 when compared to all other groups. Data are representative of at least two independent experiments. Survival curves were determined to be significantly different (p < 0.0001) by Log-rank (Mantel-Cox) test. ΔWU2 = heat-killed WU2.

**Figure 2:**
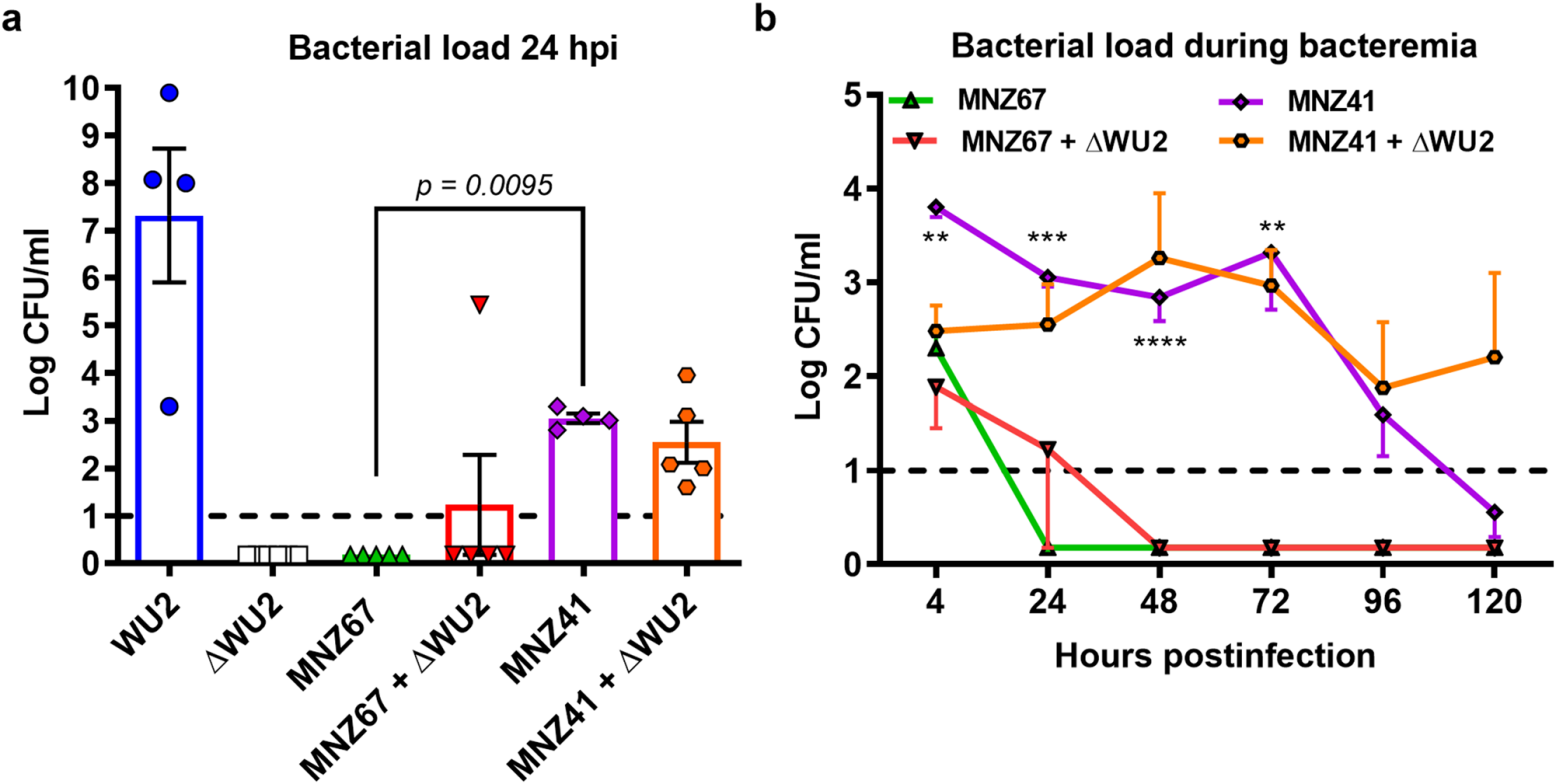
Pneumococcal CFUs recovered from the blood of infected mice. Seven- week-old mice were challenged with 10^8^ colony forming units (CFUs). Bacterial loads at 24 h post infection (**a**) and during the indicated time course (**b**) were estimated by plating blood mixtures on blood agar plates. At 24 hpi, significantly higher bacterial loads (*p* < 0.01) were recovered from MNZ41-infected mice compared to MNZ67-infected mice (One-Way ANOVA with Tukey’s post-hoc analysis). From 4–72 hpi, MNZ41-infected mice had significantly higher (*p* < 0.01) bacterial loads compared to MNZ67-infected mice regardless of co-administration of heat-killed WU2 (Two-Way ANOVA with Tukey’s post-hoc analysis). Data shown represent at least two independent experiments. Dashed line indicates the limit of detection. Error bars represent standard error of the means. **p < 0.01, ***p < 0.001, ****p < 0.0001 vs MNZ67.

Large variances in bacterial burden occurred in mice that were infected with NESp strain MNZ67 and co-administered ΔWU2. In half of the mice administered MNZ67 and ΔWU2, MNZ67 successfully acquired capsule that was verified by culture methods and PCR analysis. In this subset of mice, bacterial burdens were high and resulted in 100% mortality by 48 hpi (Fig. 1). In the remaining half of the mice administered MNZ67 and ΔWU2, there was no evidence of in vivo capsule acquisition. In this subgroup of mice, MNZ67 was rapidly cleared and all mice survived, resulting in an overall mortality of 50% by 48 hpi (Figs. 1 and 2). In mice infected with NESp strain MNZ41, bacterial loads were significantly higher (*p* < 0.01) compared to MNZ67-infected mice from as early as 4 hpi through 72 hpi (Fig. 2). Importantly, MNZ41 capsule acquisition did not significantly enhance murine mortality or bacterial burden when compared to the parent strain (Figs. 1 and 2). Furthermore, NESp MNZ41 was not rapidly cleared during systemic infection (Fig. 2b). However, NESp MNZ41 were cleared from infected mice by 120 hpi while capsule-transformed MNZ41 continued to be isolated from MNZ41-infected mice at 120 hpi (Fig. 2b).

### NESp strains acquire virulence factors from an encapsulated strain during bacteremia

**Figure 3:**
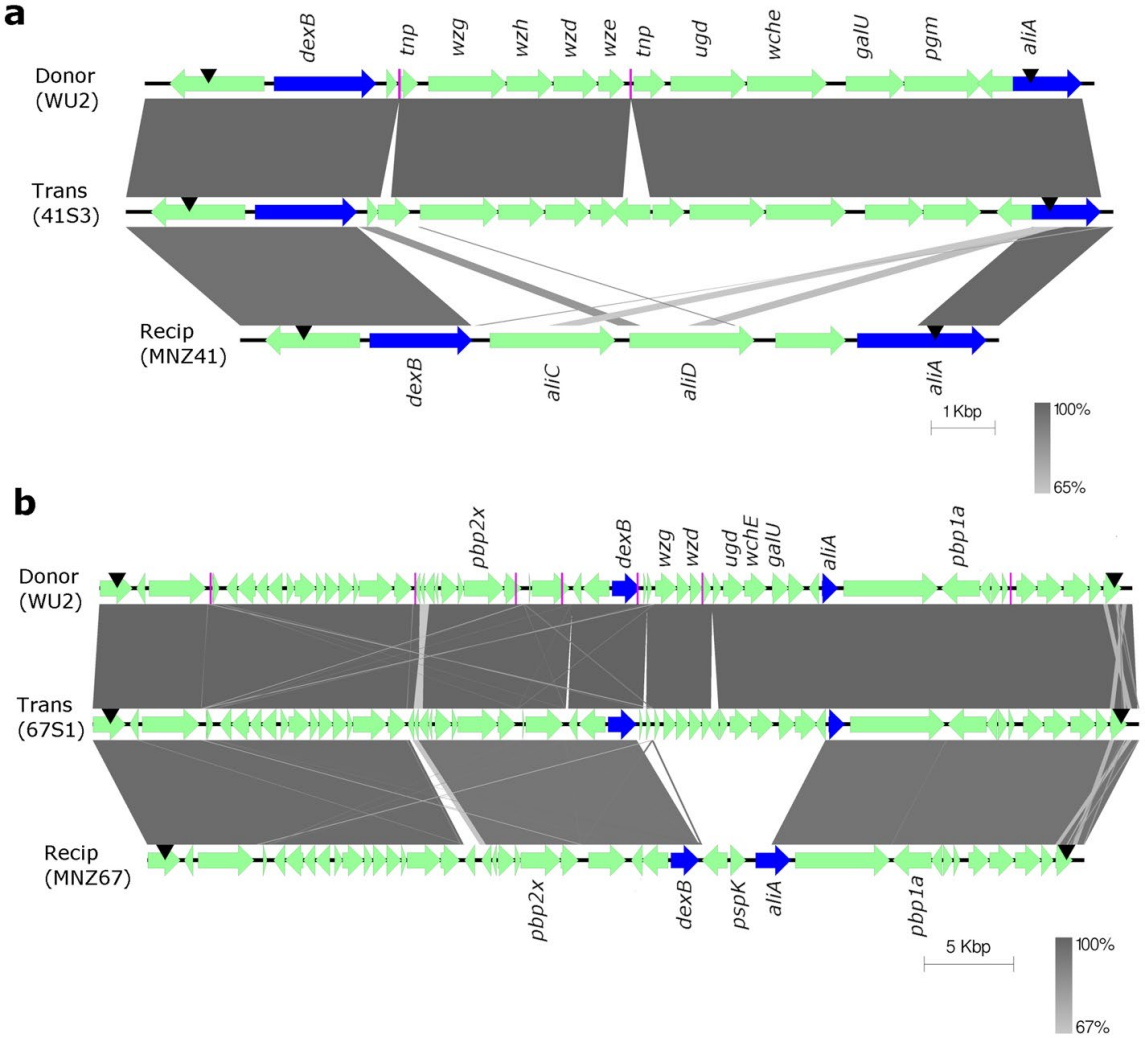
Genomic comparison of recombination regions involving capsule locus among donor, transformant, and recipient. For both recombination events (**a**, **b**), encapsulated serotype 3 *S. pneumoniae* strain WU2 served as the donor strain. (**a**) NESp strain MNZ41 is the recipient and 41S3 is the transformant. (**b**) NESp strain MNZ67 is the recipient and 67S1 is the transformant. Trans = Transformant, Recip = Recipient. Arrows indicate direction of open reading frame (ORF). Blue arrows indicate either *dexB* or *aliA* capsule-flanking genes to demarcate the capsule region. Magenta lines represent contig breakpoints. Black pointers represent recombination breakpoints. Grayscale represents % nucleotide identity between sequences.

The recombination events involving the capsular locus, and the potential for other recombination events outside the capsular locus, were examined by whole genome sequencing. The sequence types of smooth transformants 67S1 and 41S3 matched the nonencapsulated parent strains (Table 1), thus confirming the background of the transformants. A single recombination event was identified in the genome of transformant 41S3. The breakpoints of this event occurred within a gene adjacent to *dexB* and within the *aliA* gene, and resulted in the replacement of a 9.9 kbp fragment with a 13.5 kbp fragment that included the serotype 3 capsular genes (Fig. 3a). In contrast, three recombination events were identified in the genome of transformant 67S1. The first event was the replacement of a 50.5 kbp fragment with a 56.7 kbp fragment that included the serotype 3 capsular genes as well as the nearby *pbp1a* and *pbp2x* genes (Fig. 3b) that confer resistance to beta-lactam antibiotics^23^. The other two recombination events identified in 67S1 were distant to the capsular locus: one event of 4.9 kbp (Fig. S2) included the *liaFSR* genes that sense cell envelope stress^24^, and the other event of 10.2 kbp (Fig. S3) included the *nanB* gene associated with cleavage of host sialic acid^25^.

### NESp transformants express functional capsule that aids in persistence during bacteremia

**Figure 4:**
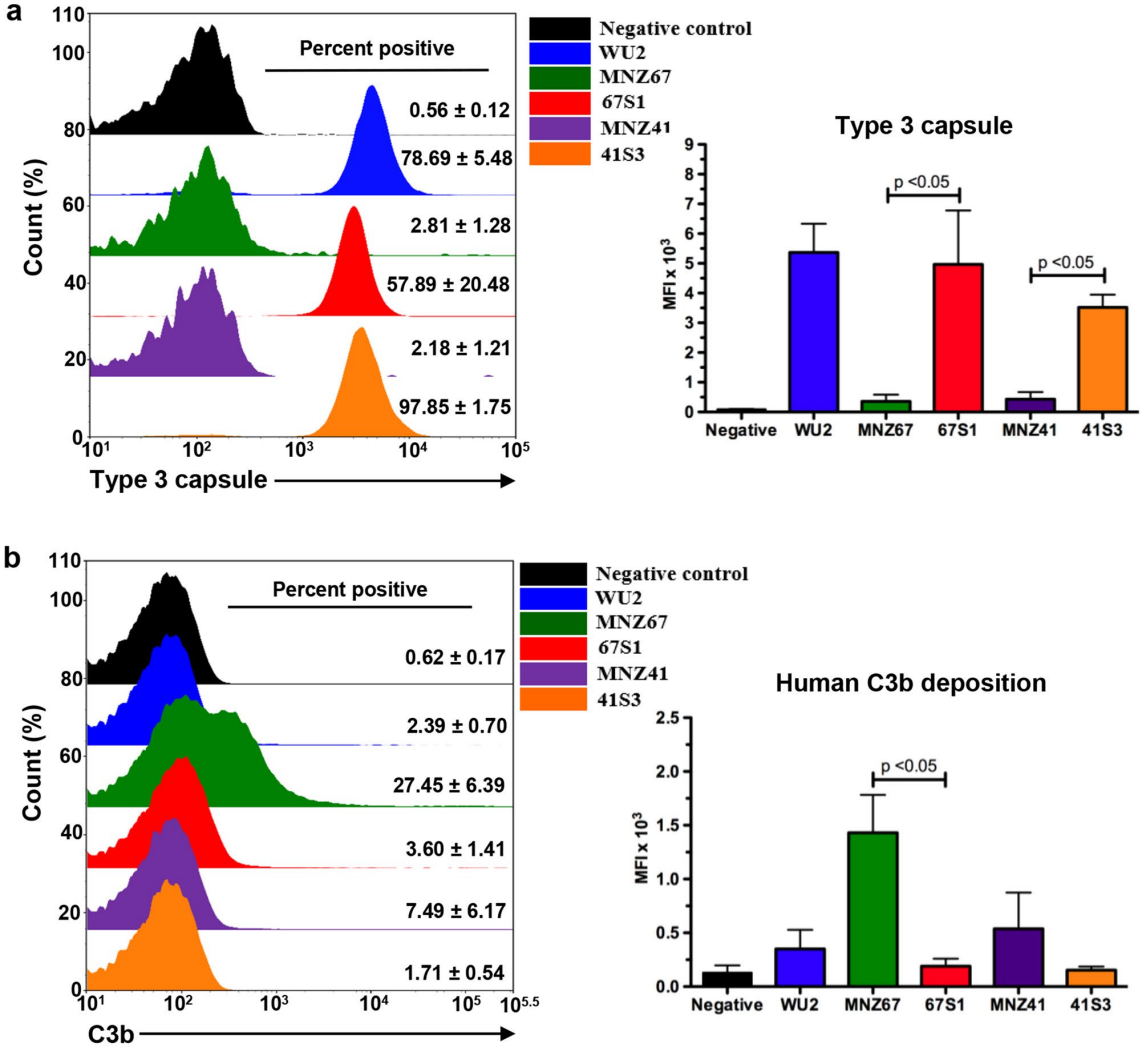
Flow cytometry evaluating functional capsule production and deposition of C3b on the surface of viable pneumococci. Pneumococci were incubated with antibodies against Type 3 capsule (**a**) or normal human serum followed by antibodies against complement component C3b (**b**). Cells were subsequently stained with Alexa Fluor 488 and subjected to flow cytometry. Percent positive cells were calculated by gating fluorescence higher than background (black line = gate), and averages of percent positive cells ± standard error of the mean are listed to the right of representative histograms in overlays. Mean fluorescent intensities (MFIs) are represented in bar graphs. Data represent at least two independent experiments with 100,000 events collected for each sample. Error bars denote standard error of the mean.

Flow cytometry was used to verify functional capsule production in 67S1 and 41S3 transformants. Encapsulated transformants bound to antibodies specific to the Type 3 capsule (Fig. 4a), indicating functional capsule production. We then wanted to determine if the capsule was providing NESp with a protective effect by shielding pneumococci from immune effectors present in the blood. The main mechanism of clearance during bacteremia is activation of the host innate complement system and deposition of C3b on the surface of invading pneumococci, which results in subsequent opsonophagocytosis by immune cells^26,27^. The amount of C3b deposited on the surface of MNZ67 was significantly greater (*p* < 0.05) than the amount of C3b deposited on the surface of the capsule transformant 67S1 (Fig. 4b). Contrarily, there was no significant difference between the amount of C3b deposited on the surface of MNZ41 when compared to its capsule transformant 41S3 (Fig. 4b).

## Discussion

The main findings of this study were that NESp strains are highly transformable and able to acquire large DNA segments that enhance NESp persistence and virulence during invasive disease. When compared to rigorously optimized transformation protocols for encapsulated pneumococci that resulted in a 1/1000 transformation frequency^28^, both carriage isolates MNZ67 and MNZ41 were highly transformable with approximately 1 positive transformant identified out of every 500 viable cells. During pneumococcal transformation, natural competence is induced through a quorum-sensing mechanism involving peptide pheromones that have been identified as two isoforms, competence-stimulating peptide (Csp)-1 and Csp-2^29,30^. MNZ67 required only Csp-1 while MNZ41 required both Csp-1 and Csp-2 for transformation. Based on sequence analysis searching for disruptions or duplications of competence genes, there was no potential explanation for the necessity of both Csp isoforms during MNZ41 transformation. However, MNZ41 encodes *aliC* and *aliD*, which are oligopeptide binding lipoproteins (OBLs) and homologues of *aliA* and *aliB*^9,31^. AliA, AliB, and AliC have been shown to impact competence induction, though the mechanism has not been fully elucidated^30,32^. It is possible that oligopeptide import involving these OBLs could be altering the quorum sensing or downstream signaling events that occur during competence induction. Further studies are needed to fully understand the impact of imported oligopeptides on the natural competence of pneumococci.

Moreover, capsule expression corresponded with bacterial burden and mortality during murine bacteremia. MNZ67 was rapidly cleared when there was no evidence of capsule acquisition in vivo. However, high mortality was associated with mice infected with capsule-transformed MNZ67. Nonetheless, capsule expression by transformed MNZ41 did not have as large an impact on MNZ41 virulence. MNZ41 was able to replicate in the blood through 4 dpi before being cleared; however, capsule acquisition did permit persistence of transformed MNZ41 in the blood for a longer duration. This result was consistent with our previous demonstration that MNZ41 can replicate in whole blood and resist immune effectors^33^. Furthermore, we have previously shown that MNZ41 expresses a choline binding protein variant that counteracts complement deposition^33^. Thus, it seems MNZ41 has already evolved virulence mechanisms that compensate for the lack of a protective capsule. Contrarily, encapsulation of MNZ67 transformant 67S1 significantly decreased C3b deposition, suggesting an increased pathogenic potential of the transformed MNZ67 strain. Importantly, all randomly sampled colonies isolated during bacteremia were resistant to the same antibiotics as parent NESp strains (Table 1). Therefore, NESp were able to acquire capsule and maintain antibiotic resistance during in vivo transformation.

Enhanced virulence and mortality associated with transformed MNZ67 could also be attributed to the recombination events occurring in the strain. Transfer of a 56.7 kbp fragment into the capsular locus of MNZ67 is a recombination event length at the upper end of what has been reported for the pneumococcus^34^. This event included recombination of beta-lactam resistance genes encoded adjacent to the capsular locus. Additionally, recombination events identified outside of the capsule locus included genes associated with cell envelope stress and cleavage of host sialic acid by a pneumococcal neuraminidase, which could also aid in persistence and enhanced virulence of this strain. Notably, the pneumococcal neuraminidase NanA has become an increasingly popular vaccine candidate^35^. However, we provide evidence that recombination involving the *nanA* locus occurs under conditions modeling a systemic infection.

Overall, these findings support our hypothesis that NESp can acquire capsule from an encapsulated strain during bacteremia and transform into antibiotic resistant and more virulent strains. As nonencapsulated and encapsulated pneumococci continue to intimately interact during colonization, the potential for emergence of recombined strains will continue to increase. Current vaccine and treatment strategies will further select these strains that threaten treatment and survival outcomes. Surprisingly, capsule acquisition did not enhance virulence of strain MNZ41. Thus, our findings also indicate that NESp have acquired virulence mechanisms that compensate for lack of capsule and permit persistence during invasive disease.

## Materials and methods

### Bacterial growth and antibiotic susceptibility

*S. pneumoniae* strains were grown at 37 °C with 5% CO_2_ on blood agar (BA) containing 5 µg/ml gentamycin or in Todd-Hewitt broth supplemented with 0.5% yeast extract (THY). To determine antibiotic susceptibility, bacteria were grown on BA containing 50 µg/ml trimethoprim (Tmp), 0.3 µg/ml erythromycin (Erm), or 100 µg/ml streptomycin. A table of strains used in this study and relative phenotypic characteristics of strains are listed in Table 1.

### In vitro transformation of NESp

Pneumococci were preconditioned in competence media (10 ml THY supplemented with 500 µl of 4% bovine serum albumin, 100 µl of 20% glucose, and 20 µl of 10% CaCl_2_) by culturing cells to mid-log phase (OD_600_ ~ 0.2) at 37 °C with 5% CO_2_. Preconditioned cultures were diluted 1:100 in fresh competence media and incubated with 2 µl of 0.1 mg/ml competence-stimulating peptide-1 (Csp-1), competence-stimulating peptide-2 (Csp-2), or both Csp-1 and Csp-2 for 12 min in a 37 °C water bath. Donor DNA (500 ng genomic LEK06 prepared with Qiagen DNeasy kit) was added to 100 µl of stimulated cells and incubated in a 30 °C water bath for 20 min. After allowing contact of exogenous DNA and bacteria, mixtures were diluted 1:100 in fresh competence media and allowed to expand in a 37 °C water bath for 1.5 h before plating on selective BA. Plates were incubated overnight at 37 °C with 5% CO_2_ before selecting transformants.

### Murine systemic infection

Seven-week-old C57BL/6 mice were injected intraperitoneally with 10^8^ colony forming units (CFUs) of serotype 3 encapsulated strain WU2, heat-inactivated WU2 (ΔWU2), nonencapsulated *S. pneumoniae* (NESp) strain MNZ67, NESp strain MNZ41, or a combination of ΔWU2 with MNZ41 or MNZ67. Blood samples were collected at 4 h postinfection, followed by sampling every 24 h until 8 days postinfection. Blood mixtures were serially diluted and plated on BA to enumerate pneumococcal CFU and determine recovered phenotypes. Up to ten random colonies from each plated blood sample were quadrant streaked onto selective blood agar plates to determine antibiotic susceptibility. Ten capsule-transformed colonies (five from MNZ41-infected mice and five from MNZ67-infected mice) were expanded in THY broth and saved for downstream analysis. Murine physical conditions and survival was monitored throughout experimental bacteremia by licensed veterinarians. Animal procedures were performed in accordance with protocols reviewed and approved by the Institutional Animal Care and Use Committee of the University of Mississippi Medical Center.

### Polymerase chain reaction (PCR) amplification

PCR amplification was used to determine the presence or absence of genes located in the capsule polysaccharide biosynthetic (*cps*) locus with primer sets previously published^36^. Genomic DNA was isolated using a DNeasy kit (Qiagen). To verify capsule gene acquisition, primers specific for the conserved gene *cpsA* were used, and WU2 genomic DNA served as a positive control for *cpsA* amplification. Primers specific to NESp genes *pspK*, *aliC*, and *aliD* were also used to determine if capsule acquisition resulted in loss of NESp genes encoded in the same locus as *cpsA*. MNZ67 genomic DNA was used as a positive control for *pspK* amplification, and MNZ41 genomic DNA was used as a positive control for *aliC* and *aliD* amplifications. Nuclease-free water was added in place of template as a negative control for amplification using all primer sets. PCR products were amplified using GoTaq DNA polymerase (Promega) with an annealing temperature of 52 °C and cycling parameters recommended by Promega. Relative sizes of the PCR products were verified using gel electrophoresis and ethidium bromide staining.

### Genome sequencing and analysis

Encapsulated donor strain WU2 and NESp recipient strain MNZ41 were previously sequenced, and available reads and assemblies were downloaded from public databases: BioProject accessions PRJEB3084 (ERR326358) for WU2 and PRJNA196249 for MNZ41. NESp recipient strain MNZ67 and capsule transformants 41S3 and 67S1 were sequenced on an Illumina MiSeq as described previously^37^ with the exception that 300 bp paired-end libraries were sequenced. The new sequences were assigned BioProject accession PRJNA450976. Sequences were adapter and quality-trimmed with BBMap^38^, and assembled de novo as done previously^37^. MAUVE^39^ v2.3.1 was used to align and reorder the contigs of the assemblies of the recipient strains to the completely sequenced NESp reference strain 110.58^40^ (GenBank accession CP007593). The reads from the donor, recipients, and transformants were then mapped to the reordered contigs of the recipients with BWA^41^ v0.7.12, coordinate-sorted and deduplicated with Picard^42^ v1.141, and realigned around short insertion-deletion polymorphisms (indels) with GATK^43^ v2.8–1. Variants were called with the UnifiedGenotyper walker of GATK, and biallelic single nucleotide polymorphisms (biSNPs) were quality-filtered with BCFtools^44^ v1.9. Recombination events were characterized with a two-step procedure that included: (1) identification of the events by following biSNPs in the core genome that are shared among the donor, recipients, and transformants, and (2) blastN alignment of the corresponding portions of the assemblies, which includes accessory sequences that are not shared by all strains, and visualization with Easyfig^45^ v2.2.2. Three or more consecutive biSNPs where the donor and transformants shared the same alternate allele compared to the recipients were considered to be part of the same recombination event. Those biSNPs identified from the self-mapping of recipient reads against the recipient assemblies were considered to be ambiguous and ignored in determining consecutive biSNPs. This analysis was performed separately with the sequences of the two in vivo transformation experiments.

### Flow cytometry

Flow cytometric analysis was used to confirm functional capsule production and evaluate phenotypic variances occurring after capsule acquisition in transformants. In all experiments, 10^7^ pneumococci were grown to mid-log phase, collected by centrifugation, and washed with phosphate-buffered saline (PBS). To evaluate capsule production, pneumococci were incubated on ice for 30 min with 200 µl monoclonal mouse anti-Type 3 capsule antibody Hyp3M6 (IgM isotype, diluted 1:20, kindly provided by Dr. Moon Nahm, University of Alabama at Birmingham, Birmingham, Alabama, USA). Bacterial cells were then collected by centrifugation, washed three times with PBS, suspended in 200 µl of 1 µg/ml biotinylated goat anti-mouse IgM antibody (Southern Biotech), and incubated on ice for 30 min. Cells incubated with PBS instead of Type 3 capsule antibody served as the negative control. To evaluate complement C3b deposition, pneumococci were incubated on ice for 30 min with 10% pooled normal human serum diluted in gelatin-based veronal buffer (0.15 mM CaCl_2_, 141 mM NaCl, 0.5 mM MgCl_2_, 0.1% gelatin, 1.8 mM sodium barbital, and 3.1 mM barbituric acid, pH 7.3–7.4). Pneumococci were subsequently collected, washed three times with PBS, suspended in 200 µl of 1 µg/ml biotinylated anti-human C3b antibody (Cedarlane Laboratories Limited), and incubated on ice for 30 min. Cells incubated with PBS instead of human serum served as the negative control. For all experiments, pneumococci were stained with streptavidin-conjugated Alexa Fluor 488 (1:1000, Invitrogen) and incubated on ice for 30 min in the dark. After staining, pneumococci were collected, washed four times with PBS, suspended in 500 µl PBS, and analyzed by a NovoCyte flow cytometer with NovoSampler (ACEA Biosciences, Inc).

### Statistics

Results were analyzed using PRISM 8 software (GraphPad Software, Inc). A student t-test was used to determine differences in mean transformation efficiencies comparing two strains. One-way analysis of variance (ANOVA) was used to evaluate if there were any overall significant differences in the means between all compared groups. Tukey posttests were performed to specifically identify the significant differences in the groups analyzed. For analysis of variance in bacterial populations over a time course, a Two-way ANOVA with Tukey posttest was performed. A p-value less than 0.05 was considered to be statistically significant.

## Supplementary information

Supplementary Information

## Data Availability

Genome sequences can be accessed with accession numbers provided in the sequencing methods. The remaining data that support the findings of this study are available upon request to the corresponding author.
