# Supplementary material for: Transformation of nonencapsulated Streptococcus pneumoniae during systemic infection
Source: Sci Rep. 2020 Nov 3;10:18932. doi: 10.1038/s41598-020-75988-5 (PMC7641166; doi:10.1038/s41598-020-75988-5)
Supplement: Supplementary file 1 — Supplementary Information [file 41598_2020_75988_MOESM1_ESM.docx]

**Transformation of Nonencapsulated *Streptococcus* *pneumoniae* During Systemic Infection**

Jessica L. Bradshaw, Iftekhar M. Rafiqullah, D. Ashley Robinson, and Larry S. McDaniel

Department of Microbiology and Immunology, University of Mississippi Medical Center, Jackson, MS, USA

**
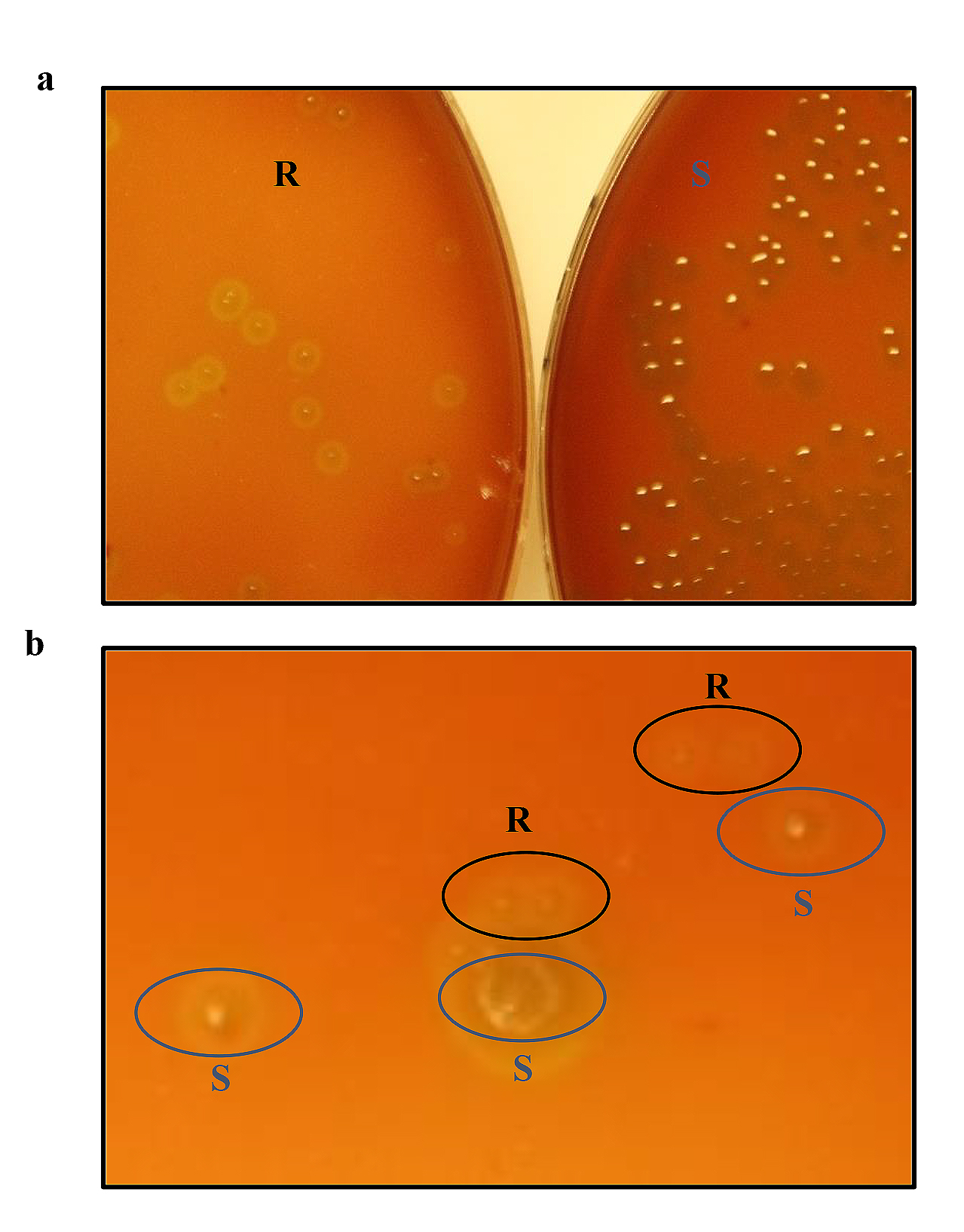
**

**Figure S1. Representative pneumococcal colonies isolated from murine blood at 24 hours post infection.** a) Blood agar plates displaying growth of homozygous rough NESp MNZ41 colonies on the left and smooth transformants on the right isolated from separate mice. b) Mixed isolation of rough and smooth colonies from an individual mouse. R = rough phenotype (nonencapsulated). S = smooth phenotype (encapsulated transformants).

**
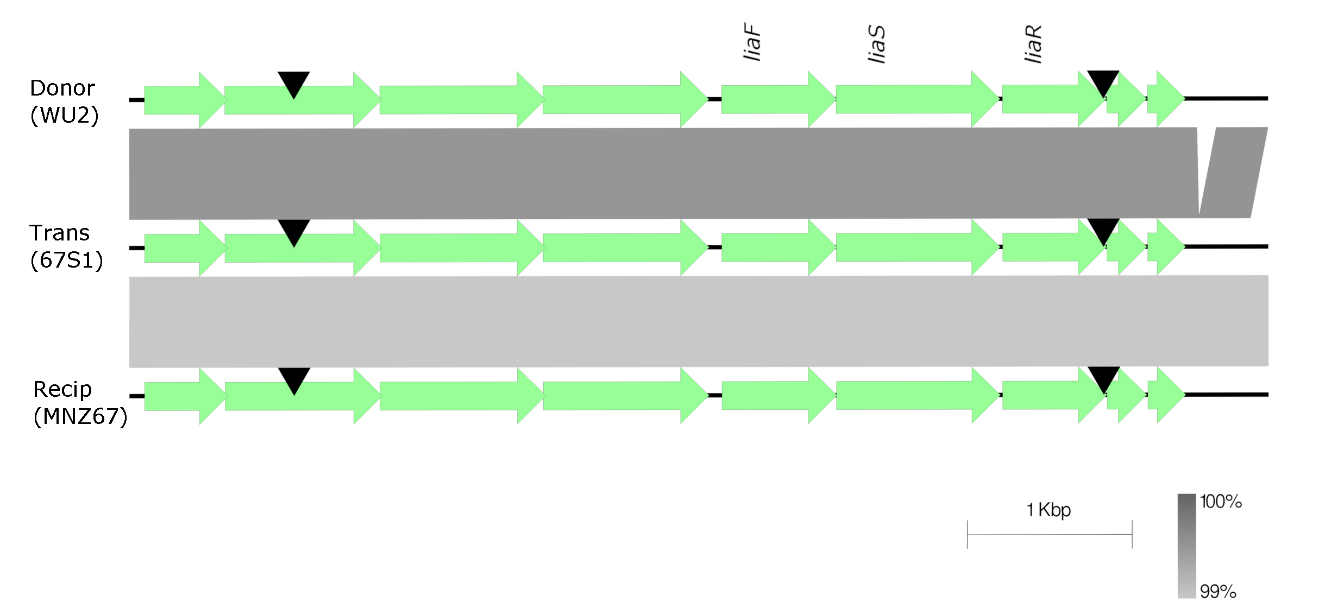
**

**Figure S2. Genomic comparison of 4.9 kbp recombination region among donor, transformant and recipient.** Encapsulated serotype 3 *S. pneumoniae* strain WU2 served as the donor strain, NESp MNZ67 was the recipient strain, and 67S1 was the transformant. Genes *liaFSR* are present within recombination region. Trans = Transformant, Recip = Recipient. Arrows indicate direction of open reading frame (ORF). Black pointers indicate recombination breakpoints. Grayscale represents % nucleotide identity between sequences.

**
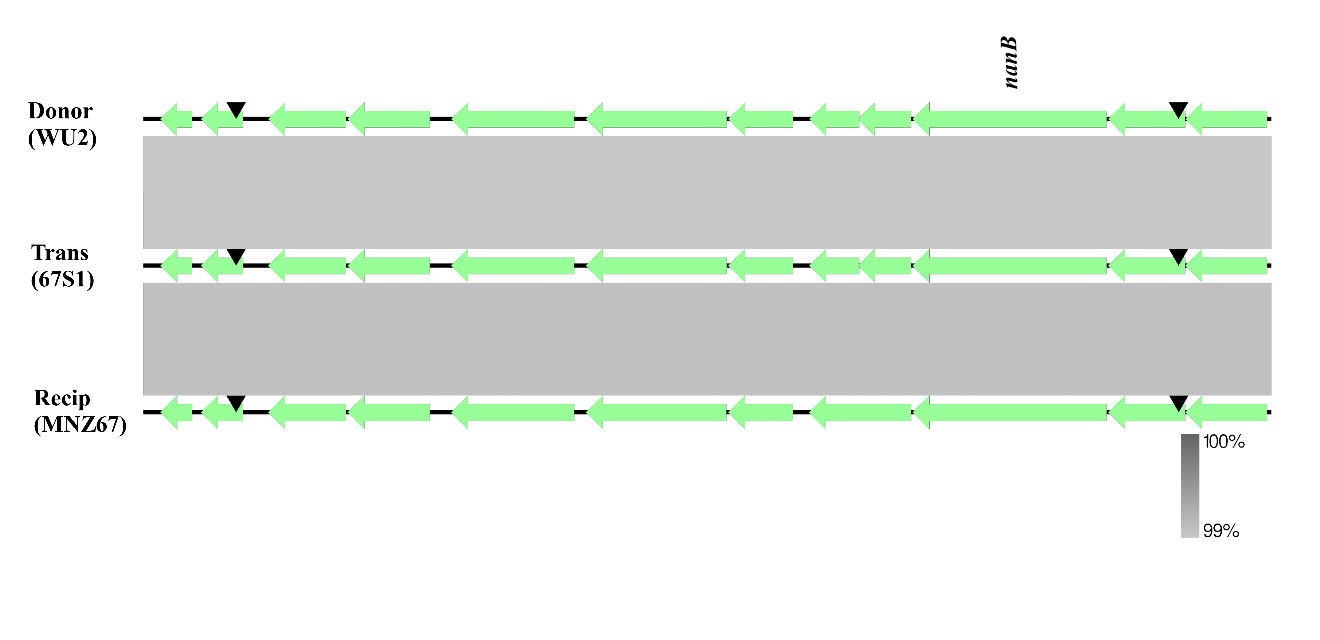
**

**Figure S3. Genomic comparison of 10.2 kbp recombination region among donor, transformant and recipient.** Encapsulated serotype 3 *S. pneumoniae* strain WU2 served as the donor strain, NESp MNZ67 was the recipient strain, and 67S1 was the transformant. Gene *nanB* is present within the recombination region. Trans = Transformant, Recip = Recipient. Arrows indicate direction of open reading frame (ORF). Black pointers indicate recombination breakpoints. Grayscale represents % nucleotide identity between sequences.
